# Association of Preterm Singleton Birth With Fertility Treatment in the US

**DOI:** 10.1001/jamanetworkopen.2021.47782

**Published:** 2022-02-08

**Authors:** Ran Wang, Qiqi Shi, Bing Jia, Wenbo Zhang, Huifeng Zhang, Yaping Shan, Linxia Qiao, Gang Chen, Chao Chen

**Affiliations:** 1 Department of Neonatology, Children’s Hospital of Fudan University, National Children’s Medical Center, Shanghai, China; 2 Department of Pediatric Cardiothoracic Surgery, Children’s Hospital of Fudan University, National Children’s Medical Center, Shanghai, China; 3 Kunshan Maternity and Children's Health Care Hospital, Kunshan, China

## Abstract

**Importance:**

Preterm birth is a global health issue. The association between fertility treatment and preterm singleton births has not been clarified.

**Objective:**

To examine the association between fertility treatment and preterm birth.

**Design, Setting, and Participants:**

This population-based cohort study used birth data that were submitted by 50 states and the District of Columbia to the National Vital Statistics System database. All mothers in the database who had a singleton live birth from January 1, 2016, to December 31, 2019, were included. Those who had preexisting hypertension or diabetes were excluded.

**Exposures:**

Fertility treatment categorized as assisted reproductive technology (ART) or non-ART treatment.

**Main Outcomes and Measures:**

The main outcome was a diagnosis of preterm birth, which was defined as birth before 37 complete weeks (<259 days) of gestation. Gestational age was calculated by obstetric estimation at delivery and was collected from the database.

**Results:**

The final sample consisted of 14 370 920 mothers (mean [SD] age, 28.79 [5.79] years) with singleton live births. Of these women, 122 944 (0.9%) conceived by ART and 71 176 (0.5%) received non-ART treatment. The prevalence of preterm birth was 7.6% (n = 1 071 994) in natural conception, 10.7% (n = 13 205) in ART, and 9.3% (n = 6629) in non-ART groups. Compared with neonates who were naturally conceived, newborns who were conceived with ART (adjusted risk difference [aRD], 3.10% [95% CI, 2.93%-3.27%]; adjusted odds ratio [aOR], 1.49 [95% CI, 1.46-1.52]; *P* < .001) and non-ART treatment (aRD, 2.22% [95% CI, 2.00%-2.44%]; aOR, 1.35 [95% CI, 1.31-1.38]; *P* < .001) had significantly higher risk for preterm birth after full adjustment. These associations were similar in subgroups of participants as defined by baseline characteristics.

**Conclusions and Relevance:**

This study found that singleton neonates who were conceived by fertility treatment had higher rates of preterm birth. Further investigations are warranted into the association between ART or non-ART treatment and the risk of preterm birth in singleton neonates.

## Key Points

**Question:**

Are singleton pregnancies with fertility treatment associated with an increased risk for preterm birth?

**Findings:**

In this cohort study of 14 370 920 mother-newborn pairs, fertility treatment, including assisted reproductive technology and other procedures, was associated with preterm singleton births. Neonates who were conceived by fertility treatment had higher rates of very preterm and extremely preterm birth.

**Meaning:**

The findings suggest an association between fertility treatment and preterm birth in singleton-birth neonates, warranting additional investigation.

## Teaser

This cohort study compares the risk of preterm birth among mothers with singleton live births who conceived naturally and those who used assisted reproductive technology or other fertility treatment.

## Introduction

Preterm birth is a crucial global health issue. The worldwide incidence of preterm birth was 10.6%, equating to 14.84 million live-born preterm babies in 2014,^1^ and the incidence did not decrease in recent years despite the advances in perinatal care. Prematurity and its associated complications are the leading cause of death in neonates and children younger than 5 years worldwide.^2,3^ Furthermore, preterm neonates are at a higher risk for lifelong health and developmental challenges compared with term newborns.^4,5^ It is imperative to identify the essential risk factors for preterm birth and to enhance perinatal and neonatal management.

Infertility, defined as the failure to achieve pregnancy after 12 months of regular unprotected sexual intercourse, affects 12.1% of US women aged 15 to 49 years^6^ and 8% to 12% of the childbearing population worldwide.^7,8^ The World Health Organization recognizes infertility as a global public health issue.^9^ With the rapid development of fertility treatment, which includes assisted reproductive technology (ART)^10^ and non-ART medically assisted treatment, conceiving a child has been possible for infertile couples. Assisted reproductive technology is defined as treatment in which eggs or embryos are handled for the purpose of achieving pregnancy. In the US, the number of fertility treatments and fertility treatment–conceived births has increased steadily since the early 1980s.^11,12^ At present, approximately 2% of pregnancies are conceived through ART, and its use has been increasing in recent years.^13,14^ Non-ART treatment, which includes fertility-enhancing drugs, artificial insemination, and intrauterine insemination, accounted for even a higher proportion of pregnancies.^15^ Although greater availability of fertility treatments can help reduce the burden of infertility worldwide, some studies found that fertility treatments were associated with an increased risk of adverse health outcomes for both mothers and neonates, including preterm birth.^16^

A consequence of the increased use of fertility treatment, especially ART, is a substantially higher rate of multiple pregnancies.^14^ Compared with singleton pregnancies, multiple pregnancies may be at greater risk for preterm birth. The association between fertility treatment and preterm birth in singleton neonates has not been clarified. Most studies have suggested that even singleton neonates who were conceived with ART may also be at higher risk for preterm delivery compared with neonates in the general population.^17,18^ However, a prospective cohort study published in 2020 found no substantial difference in adverse outcomes associated with ART in singleton pregnancies.^19^ Furthermore, the association between non-ART treatment and preterm delivery has not been comprehensively investigated.

The association between fertility interventions and preterm birth needs to be explored in a large population with substantial available information for potential confounding factors to enable physicians to provide tailored prepregnancy and postpregnancy advice and care to women undergoing fertility treatment. In this cohort study, we used US birth certificate data to examine the association between fertility treatment and preterm birth.

## Methods

### Study Population

This prospective, comparative, population-based cohort study used the National Vital Statistics System, a database that contains birth and death records that are electronically submitted by the 50 states and District of Columbia to the Centers for Disease Control and Prevention and National Center for Health Statistics. All US states had implemented the revised birth certificate as of January 1, 2016. Specifically, for this study, we obtained maternal and neonatal demographic and health characteristics for all mothers in the database with singleton live births from January 1, 2016, to December 31, 2019, excluding those with prepregnancy hypertension or diabetes. Records with no information on gestational age or fertility treatment were also excluded. The Children’s Hospital of Fudan University Institutional Review Board deemed the study exempt from review and waived the informed consent requirement because the data used were publicly available and the study did not involve human participants. We followed the Strengthening the Reporting of Observational Studies in Epidemiology (STROBE) reporting guideline.

### Outcome and Fertility Treatment Exposure

The main outcome was diagnosis of preterm birth, which was defined as birth before 37 complete weeks (<259 days) of gestation according to the *International Classification of Diseases, Ninth Revision* and *International Statistical Classification of Diseases and Related Health Problems, Tenth Revision*. Gestational age was calculated by obstetric estimation at delivery and was collected from the database. We subdivided the outcome into 3 types of preterm birth: extremely (delivery <28 weeks), very (delivery at 28-31 weeks and 6 days), and moderately and late (delivery at 32-36 weeks and 6 days). Birth weight was collected directly from the database and was converted from pounds and ounces to grams by the National Center for Health Statistics.

Exposure measurement in this study was pregnancy that resulted from fertility treatment, including (1) ART, such as in vitro fertilization, gamete intrafallopian transfer, and zygote intrafallopian transfer, and (2) non-ART treatment, such as fertility-enhancing drugs, artificial insemination, and intrauterine insemination. Mothers who received both ART and non-ART treatment were classified under the ART group. The exposure information was collected directly from the medical and health information section of the US Standard Certificate of Live Birth.

### Covariates

Variables that were considered as potential confounding factors included maternal age, race and ethnicity, educational level, marital status, parity, smoking status before pregnancy and during pregnancy, history of preterm delivery, history of cesarean delivery, prepregnancy body mass index (BMI), timing of initiation of prenatal care, gestational hypertension, eclampsia, gestational diabetes, and neonate sex.

Maternal age was defined as age at the time of birth and classified as younger than 30, 30 to 34, 35 to 39, or 40 years or older. Maternal race and ethnicity were self-reported in the database and included Hispanic, non-Hispanic Black, non-Hispanic White, and other individuals (which included non-Hispanic Asian, non-Hispanic Native American or Alaska Native, non-Hispanic Native Hawaiian or Other Pacific Islander, non-Hispanic people of more than 1 race, people of unknown racial or ethnic origin, or those who did not state race and ethnicity). Maternal educational levels were recorded as lower than high school diploma, high school diploma, or higher than high school diploma. Marital status was categorized as married or unmarried. Parity, defined as the total number of live births excluding the current delivery, was classified as 0, 1, 2, 3, or 4 or more. Smoking status before pregnancy and during pregnancy was classified as yes or no. Time of initiation of prenatal care was categorized by the trimester of the first prenatal visit as no prenatal care, first to third month, fourth to sixth month, and seventh to ninth month. Maternal prepregnancy BMI was calculated as prepregnancy weight in kilograms divided by height in meters squared and classified as less than 18.5, 18.5 to 24.9, 25.0 to 29.9, 30.0 to 34.9, 35.0 to 39.9, or 40 or higher. Gestational diabetes, gestational hypertension, and eclampsia were each categorized as yes or no. History of preterm delivery was classified as yes or no. These maternal variables were identified from the facility worksheet of the US Standard Certificate of Live Birth.

Neonate sex was categorized as male or female. Birth weight was converted from pounds and ounces to grams by the National Center for Health Statistics. We subdivided birth weight into low birth weight (<2500 g), very low birth weight (<1500 g), or extremely low birth weight (<1000 g). Variables including birth weight and neonate sex were collected from the database. A missing category for covariates was added when necessary.

### Statistical Analysis

Statistical analyses were performed using Stata, version 15.0 (StataCorp LLC). All *P* values were 2-sided, and *P* < .05 was considered to be statistically significant. Means and SDs were reported for continuous variables, and frequencies and percentages were reported for categorical variables. Differences were tested using the χ^2^ test for categorical variables and an unpaired, 2-tailed *t* test or analysis of variance for numerical variables.

We performed a series of logistic regression models to estimate the odds ratios (ORs) with 95% CIs of preterm birth vs fertility treatment. In the main analyses, we estimated the association between fertility treatment (ART and non-ART treatment group separately) and preterm birth, with neonates who were conceived naturally as the reference group. Informed by previous research,^20,21,22,23,24^ we covered multiple potential confounding factors with 3 models. Model 1 was not adjusted. Model 2 was adjusted for sociodemographic characteristics, including maternal age, race and ethnicity, educational level, and marital status. Model 3 was model 2 but further adjusted for potential pregnancy-related confounders, including parity, smoking status before and during pregnancy, history of preterm delivery, history of cesarean delivery, prepregnancy BMI, timing of initiation of prenatal care, gestational hypertension, gestational diabetes, eclampsia, and neonate sex. Furthermore, all results in the main analyses were provided with an adjusted risk difference (aRD) and 95% CI using the postestimation margins command in Stata.^25^ The analyses were repeated for the finer categorization of preterm birth. We corrected for multiple testing using the Bonferroni method in the main analyses; therefore, only unadjusted *P* > .004 was considered to be significant at the 95% CI level.

In the stratified analysis, we subdivided women according to baseline characteristics (ie, maternal age, race and ethnicity, educational level, marital status, history of preterm delivery, history of cesarean delivery, prepregnancy BMI, timing of initiation of prenatal care, gestational hypertension, gestational diabetes, eclampsia, and neonate sex). Among these subgroups, we analyzed the association between fertility treatment and preterm birth after adjusting for other potential risk factors to explore potential disparities in the association between fertility treatment and preterm birth.

In addition, to examine the robustness of the results, we performed a series of sensitivity analyses. First, we reran the model after excluding potential risk factors whose effect sizes were significant in the main analyses (ie, history of cesarean delivery, history of preterm delivery, gestational hypertension, eclampsia, and gestational diabetes) to assess the implications of these complicated pregnancy conditions for the association between infertility treatment and preterm birth. Second, to examine the implication of missing covariate data for the analysis, we conducted a sensitivity analysis in which mothers with incomplete covariate data were omitted.

## Results

The final sample consisted of 14 370 920 mothers (mean [SD] age, 28.79 [5.79] years) with singleton live births (Figure). A comparison of the present sample and excluded individuals is shown in eTable 1 in the Supplement. Of these women, 3 433 864 (23.9%) had Hispanic, 2 012 563 (14.0%) had non-Hispanic Black, 7 412 132 (51.6%) had non-Hispanic White, and 1 387 539 (9.7%) had other race and ethnicity. A total of 122 944 mothers (0.9%) conceived by ART, and 71 176 (0.5%) conceived by non-ART treatment (Table 1). Compared with mothers who had natural conception (n = 14 176 800 [98.6%]), those who received ART and non-ART treatment tended to be older and were more likely to have gestational hypertension and gestational diabetes.

**Figure.**
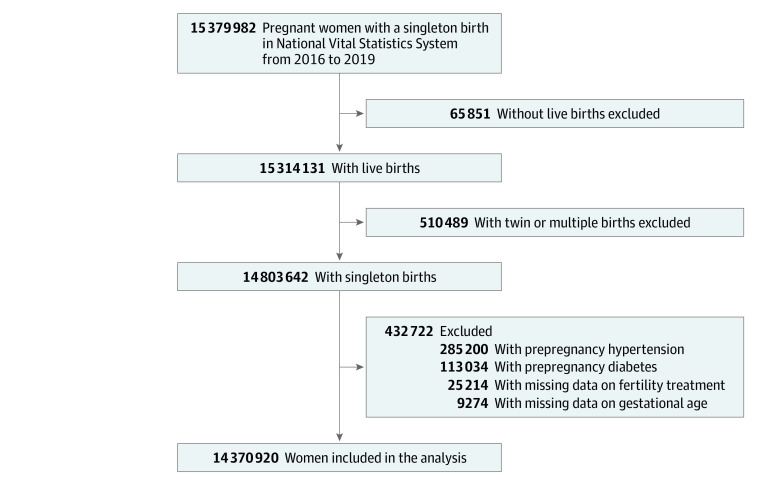
Participant Flowchart

**Table 1.** Population Characteristics by Fertility Treatment

Characteristic	No. (%)				*P* value
	All mothers	Natural conception group	Treatment group		
			ART	Non-ART	
Population					
2016-2019	14 370 920 (100)	14 176 800 (98.6)	122 944 (0.9)	71 176 (0.5)	NA
Birth year of neonate					
2016	3 704 404 (25.8)	3 662 388 (25.8)	24 790 (20.2)	17 226 (24.2)	<.001
2017	3 612 116 (25.1)	3 565 154 (25.2)	28 957 (23.5)	18 005 (25.3)	
2018	3 549 151 (24.7)	3 498 661 (24.7)	32 106 (26.1)	18 384 (25.8)	
2019	3 505 249 (24.4)	3 450 597 (24.3)	37 091 (30.2)	17 561 (24.7)	
Maternal age, mean (SD), y	28.79 (5.79)	28.71 (5.76)	35.77 (5.10)	32.65 (4.87)	<.001
Age group, y					
<30	7 809 769 (54.3)	7 779 082 (54.9)	12 117 (9.9)	18 570 (26.1)	<.001
30-34	4 080 052 (28.4)	4 012 104 (28.3)	39 776 (32.3)	28 172 (39.6)	
35-39	2 039 115 (14.2)	1 976 601 (13.9)	43 757 (35.6)	18 757 (26.3)	
≥40	441 984 (3.1)	40 9013 (2.9)	27 294 (22.2)	5677 (8.0)	
Race and ethnicity^a^					
Hispanic	3 433 864 (23.9)	3 417 569 (24.1)	10 066 (8.2)	6229 (8.7)	<.001
Non-Hispanic					
Black	2 012 563 (14.0)	2 004 204 (14.1)	5457 (4.4)	2902 (4.1)	
White	7 412 132 (51.6)	7 272 789 (51.3)	85 130 (69.2)	54 213 (76.2)	
Other^b^	1 387 539 (9.7)	1 361 665 (9.6)	18 639 (15.2)	7235 (10.2)	
Missing data	124 822 (0.9)	120 573 (0.9)	3652 (3.0)	597 (0.8)	
Educational level					
<High school diploma	1 865 360 (13.0)	1 863 172 (13.1)	1223 (1.0)	965 (1.4)	<.001
High school diploma	3 648 337 (25.4)	3 636 024 (25.7)	6625 (5.4)	5688 (8.0)	
>High school diploma	8 672 897 (60.4)	8 497 559 (59.9)	111 380 (90.6)	63 958 (89.9)	
Missing data	184 326 (1.3)	180 045 (1.3)	3716 (3.0)	565 (0.8)	
Marital status					
Married	7 833 466 (54.5)	7 669 793 (54.1)	100 613 (81.8)	63 060 (88.6)	<.001
Unmarried	5 239 894 (36.5)	5 226 471 (36.9)	8392 (6.8)	5031 (7.1)	
Missing data	1 297 560 (9.0)	1 280 536 (9.0)	13 939 (11.3)	3085 (4.3)	
Pregnancy-related characteristics					
Parity					
0	5 539 721 (38.5)	5 421 487 (38.2)	73 957 (60.2)	44 277 (62.2)	<.001
1	4 590 620 (31.9)	4 537 740 (32.0)	33 424 (27.2)	19 456 (27.3)	
2	2 429 733 (16.9)	2 415 271 (17.0)	9384 (7.6)	5078 (7.1)	
3	1 040 493 (7.2)	1 035 386 (7.3)	3576 (2.9)	1531 (2.2)	
≥4	733 148 (5.1)	729 990 (5.2)	2412 (2.0)	746 (1.1)	
Missing data	37 205 (0.3)	36 926 (0.3)	191 (0.2)	88 (0.1)	
Smoking status before pregnancy					
Yes	1 223 398 (8.5)	1 220 517 (8.6)	1260 (1.0)	1621 (2.3)	<.001
No	13 080 605 (91.0)	12 889 798 (90.9)	121 402 (98.8)	69 405 (97.5)	
Missing data	66 917 (0.5)	66 485 (0.5)	282 (0.2)	150 (0.2)	
Smoking status during pregnancy					
Yes	940 210 (6.5)	938 905 (6.6)	502 (0.4)	803 (1.1)	<.001
No	13 347 126 (92.9)	13 155 236 (92.8)	121 811 (99.1)	70 079 (98.5)	
Missing data	83 584 (0.6)	82 659 (0.6)	631 (0.5)	294 (0.4)	
Time of initiation of prenatal care					
No prenatal care	237 127 (1.7)	236 724 (1.7)	279 (0.2)	124 (0.2)	<.001
1st-3rd mo	10 822 403 (75.3)	10 650 495 (75.1)	108 421 (88.2)	63 487 (89.2)	
4th-6th mo	2 307 675 (16.1)	2 291 753 (16.2)	10 471 (8.5)	5451 (7.7)	
7th-9th mo	651 076 (4.5)	648 261 (4.6)	1763 (1.4)	1052 (1.5)	
Missing data	352 639 (2.5)	349 567 (2.5)	2010 (1.6)	1062 (1.5)	
Prepregnancy BMI, mean (SD)	28.60 (12.89)	28.62 (12.92)	26.94 (10.88)	28.30 (10.65)	
Prepregnancy BMI					
<18.5	473 894 (3.3)	469 546 (3.3)	2952 (2.4)	1396 (2.0)	<.001
18.5-24.9	6 116 404 (42.6)	6 022 982 (42.5)	62 692 (51.0)	30 730 (43.2)	
25.0-29.9	3 728 528 (25.9)	3 679 399 (26.0)	31 484 (25.6)	17 645 (24.8)	
30.0-34.9	2 058 225 (14.3)	2 033 484 (14.3)	14 424 (11.7)	10 317 (14.5)	
35.0-39.9	984 895 (6.9)	972 699 (6.9)	6251 (5.1)	5945 (8.4)	
≥40	666 225 (4.6)	658 834 (4.6)	3172 (2.6)	4219 (5.9)	
Missing data	342 749 (2.4)	339 856 (2.4)	1969 (1.6)	924 (1.3)	
Gestational hypertension					
Yes	953 269 (6.6)	932 542 (6.6)	13 293 (10.8)	7434 (10.4)	<.001
No	13 417 651 (93.4)	13 244 258 (93.4)	109 651 (89.2)	63 742 (89.6)	
Gestational diabetes					
Yes	899 405 (6.3)	879 080 (6.2)	12 784 (10.4)	7541 (10.6)	<.001
No	1 3471 515 (93.7)	13 297 720 (93.8)	110 160 (89.6)	63 635 (89.4)	
Eclampsia					
Yes	33 856 (0.2)	33 264 (0.2)	400 (0.3)	192 (0.3)	<.001
No	14 337 064 (99.8)	14 143 536 (99.8)	122 544 (99.7)	70 984 (99.7)	
History of preterm delivery					
Yes	453 146 (3.2)	447 305 (3.2)	3816 (3.1)	2025 (2.8)	<.001
No	13 917 774 (96.8)	13 729 495 (96.8)	119 128 (96.9)	69 151 (97.2)	
History of cesarean delivery					
Yes	2 182 275 (15.2)	2 156 568 (15.2)	17 507 (14.2)	8200 (11.5)	<.001
No	12 188 645 (84.8)	12 020 232 (84.8)	105 437 (85.8)	62 976 (88.5)	
Neonatal information					
Low birth weight^c^					
No	13 485 984 (93.8)	13 306 534 (93.9)	113 473 (92.3)	65 977 (92.7)	<.001
Yes	878 888 (6.1)	864 267 (6.1)	9440 (7.7)	5181 (7.3)	
Very low birth weight^c^	71 878 (0.5)	70 462 (0.5)	933 (0.8)	483 (0.7)	
Extremely low birth weight^c^	51 823 (0.4)	50 617 (0.4)	796 (0.6)	410 (0.6)	
Missing data	6048 (0)	5999 (0)	31 (0)	18 (0)	
Neonate sex					
Female	7 018 556 (48.8)	6 923 613 (48.8)	60 276 (49.0)	34 667 (48.7)	.32
Male	7352 364 (51.2)	7 253 187 (51.2)	62 668 (51.0)	36 509 (51.3)	
Preterm birth^d^					
No	13 279 092 (92.4)	13 104 806 (92.4)	109 739 (89.3)	64 547 (90.7)	<.001
Yes	1 091 828 (7.6)	1 071 994 (7.6)	13 205 (10.7)	6629 (9.3)	
Extremely preterm birth^d^	52 241 (0.4)	51 027 (0.4)	820 (0.7)	394 (0.6)	
Very preterm birth^d^	90 873 (0.6)	89 018 (0.6)	1272 (1.0)	583 (0.8)	
Moderately and late preterm birth^d^	948 714 (6.6)	931 949 (6.6)	11 113 (9.0)	5652 (7.9)	

Abbreviations: ART, assisted reproductive technology; BMI, body mass index (calculated as weight in kilograms divided by height in meters squared); NA, not applicable.

^a^ Race and ethnicity data were self-reported in the database.

^b^ Other included non-Hispanic Native American or Alaska Native, non-Hispanic Asian, non-Hispanic Native Hawaiian or Other Pacific Islander, non-Hispanic people of more than 1 race, people of unknown racial or ethnic origin, or those who did not state race and ethnicity.

^c^ Low birth weight was defined as less than 2500 g. Very low birth weight was defined as less than 1500 g. Extremely low birth weight was defined as less than 1000 g.

^d^ Preterm birth was defined as delivery before 37 weeks’ gestation. Extremely preterm birth was defined as delivery before 28 weeks’ gestation. Very preterm birth was defined as delivery at 28 to 31 weeks and 6 days’ gestation. Moderately and late preterm birth was defined as delivery at 32 to 36 weeks and 6 days’ gestation.

Of the 1 091 828 preterm births (7.6%), 948 714 (6.6%) were classified as moderately and late, 90 873 (0.6%) as very, and 52 241 (0.4%) as extremely preterm. The prevalence of preterm birth was 7.6% (n = 1 071 994) in those with natural conception, 10.7% (n = 13 205) in those who received ART, and 9.3% (n = 6629) in those who used non-ART treatment. Incidence rates of very preterm (1.0% vs 0.8% vs 0.6%; *P* < .001) and extremely preterm births (0.7% vs 0.6% vs 0.4%; *P* < .001) were significantly higher in the ART and non-ART treatment groups compared with the natural conception group (Table 1). Neonates who were conceived after ART or non-ART treatment had a higher incidence of low birth weight than those who were naturally conceived (9440 [7.7%] vs 5181 [7.3%] vs 864 267 [6.1%]; *P* < .001). Baseline characteristics are shown in Table 1.

Table 2 shows the crude and adjusted associations between fertility treatment and the risk of preterm birth. At the population level, crude analyses suggested that neonates who were conceived with the use of ART (RD, 3.18% [95% CI, 3.01%-3.35%]; OR, 1.47 [95% CI, 1.44-1.50]; *P* < .001) or non-ART treatment (RD, 1.75% [95% CI, 1.54%-1.97%]; OR, 1.26 [95% CI, 1.22-1.29]; *P* < .001) had a higher risk of preterm birth. After (model 3) adjustment for maternal age, race and ethnicity, educational level, marital status, parity, smoking status before and during pregnancy, history of preterm delivery, history of cesarean delivery, prepregnancy BMI, timing of initiation of prenatal care, gestational hypertension, eclampsia, gestational diabetes, and neonate sex, the odds of preterm birth were 49% higher in the ART group than in the natural conception group (aRD, 3.10% [95% CI, 2.93%-3.27%]; aOR, 1.49 [95% CI, 1.46-1.52]; *P* < .001) and 35% higher in the non-ART treatment group compared with the natural conception group (aRD, 2.22% [95% CI, 2.00%-2.44%]; aOR, 1.35 [95% CI, 1.31-1.38]; *P* < .001). The full logistic regression model output for all covariates is provided in eTable 2 in the Supplement.

**Table 2.** Odds Ratios and Risk Difference for the Associations Between Fertility Treatment and Preterm Birth

Model^a^	ART group		Non-ART treatment group	
	OR (95% CI)	RD (95% CI), %	OR (95% CI)	RD (95% CI), %
**Preterm birth**				
Model 1	1.47 (1.44-1.50)	3.18 (3.01-3.35)	1.26 (1.22-1.29)	1.75 (1.54-1.97)
Model 2	1.59 (1.56-1.62)	3.91 (3.73-4.10)	1.45 (1.41-1.48)	3.00 (2.76-3.24)
Model 3	1.49 (1.46-1.52)	3.10 (2.93-3.27)	1.35 (1.31-1.38)	2.22 (2.00-2.44)
**Moderately and late preterm birth**				
Model 1	1.42 (1.40-1.45)	2.56 (2.39-2.72)	1.23 (1.20-1.27)	1.41 (1.21-1.61)
Model 2	1.51 (1.48-1.54)	3.03 (2.86-3.20)	1.39 (1.35-1.43)	2.33 (2.11-2.56)
Model 3	1.46 (1.43-1.49)	2.62 (2.46-2.78)	1.32 (1.28-1.36)	1.86 (1.65-2.07)
**Very preterm birth**				
Model 1	1.71 (1.61-1.80)	0.47 (0.41-0.53)	1.33 (1.23-1.44)	0.22 (0.15-0.29)
Model 2	1.99 (1.88-2.10)	0.65 (0.58-0.73)	1.70 (1.57-1.84)	0.46 (0.37-0.56)
Model 3	1.68 (1.59-1.78)	0.44 (0.38-0.51)	1.46 (1.34-1.58)	0.30 (0.22-0.38)
**Extremely preterm birth**				
Model 1	1.86 (1.73-1.99)	0.31 (0.26-0.35)	1.54 (1.40-1.70)	0.19 (0.14-0.25)
Model 2	2.57 (2.40-2.76)	0.56 (0.49-0.62)	2.28 (2.06-2.52)	0.45 (0.37-0.53)
Model 3	1.88 (1.72-2.05)	0.22 (0.18-0.26)	1.83 (1.62-2.06)	0.20 (0.15-0.26)

Abbreviations: ART, assisted reproductive technology; OR, odds ratio; RD, risk difference.

^a^ Model 1 was not adjusted. Model 2 was adjusted for maternal age, race and ethnicity, educational level, and marital status. Model 3 was model 2 adjusted for parity, smoking status before pregnancy and during pregnancy, history of preterm delivery, history of cesarean delivery, prepregnancy body mass index, timing of initiation of prenatal care, gestational hypertension, gestational diabetes, eclampsia, and neonate sex. The natural conception group was the reference group for all models.

Further analysis was performed by a finer categorization of preterm birth. Compared with the natural conception group, the risk of very preterm birth was 68% higher in the ART group (aRD, 0.44% [95% CI, 0.38%-0.51%]; aOR, 1.68 [95% CI, 1.59-1.78]; *P* < .001) and 46% higher in the non-ART treatment group (aRD, 0.30% [95% CI, 0.22%-0.38%]; aOR, 1.46 [95% CI, 1.34-1.58]; *P* < .001) after full adjustment (model 3). Risk of extremely preterm birth increased by 88% in the ART group (aRD, 0.22% [95% CI, 0.18%-0.26%]; aOR, 1.88 [95% CI, 1.72-2.05]; *P* < .001) and by 83% in the non-ART treatment group (aRD, 0.20% [95% CI, 0.15%-0.26%]; aOR, 1.83 [95% CI, 1.62-2.06]; *P* < .001) compared with the natural conception group after full adjustment (model 3) (Table 2). These results remained statistically significant after Bonferroni correction.

Stratified analyses by maternal age, race and ethnicity, prepregnancy BMI, gestational hypertension, gestational diabetes, eclampsia, history of preterm delivery, history of cesarean delivery, and neonate sex were conducted. The neonates who were conceived with ART or non-ART treatment had a higher risk of preterm birth in almost all subgroups (Table 3).

**Table 3.** Subgroup Analyses of Associations Between Fertility Treatment and Risk of Preterm Birth

Variable^a^	aOR (95% CI)	
	ART group	Non-ART treatment group
Age group, y		
<30	1.82 (1.71-1.93)	1.48 (1.41-1.56)
30-34	1.50 (1.45-1.55)	1.29 (1.24-1.35)
35-39	1.33 (1.29-1.37)	1.26 (1.20-1.33)
≥40	1.16 (1.11-1.21)	1.13 (1.04-1.23)
Race and ethnicity^b^		
Hispanic	1.53 (1.44-1.63)	1.34 (1.24-1.46)
Non-Hispanic		
Black	1.62 (1.50-1.75)	1.53 (1.37-1.70)
White	1.52 (1.48-1.55)	1.35 (1.31-1.39)
Other^c^	1.37 (1.31-1.44)	1.29 (1.19-1.40)
Missing data	1.40 (1.25-1.57)	1.00 (0.73-1.37)
Prepregnancy BMI		
<18.5	1.58 (1.40-1.80)	1.39 (1.15-1.68)
18.5-24.9	1.52 (1.48-1.57)	1.41 (1.35-1.47)
25.0-29.9	1.53 (1.48-1.59)	1.38 (1.31-1.45)
30.0-34.9	1.47 (1.40-1.54)	1.24 (1.16-1.33)
35.0-39.9	1.31 (1.22-1.42)	1.20 (1.10-1.30)
≥40	1.25 (1.12-1.38)	1.07 (0.97-1.18)
Missing data	1.45 (1.28-1.65)	1.56 (1.29-1.88)
Gestational hypertension		
Yes	1.30 (1.25-1.36)	1.24 (1.17-1.31)
No	1.52 (1.49-1.56)	1.37 (1.33-1.41)
Gestational diabetes		
Yes	1.34 (1.27-1.41)	1.20 (1.12-1.29)
No	1.51 (1.48-1.54)	1.37 (1.33-1.40)
Eclampsia		
Yes	1.27 (1.03-1.56)	1.08 (0.80-1.46)
No	1.49 (1.46-1.52)	1.35 (1.31-1.38)
History of preterm delivery		
Yes	1.06 (0.99-1.15)	1.01 (0.91-1.13)
No	1.52 (1.49-1.55)	1.37 (1.33-1.41)
History of cesarean delivery		
Yes	1.36 (1.29-1.43)	1.24 (1.15-1.34)
No	1.52 (1.49-1.55)	1.37 (1.33-1.41)
Neonate sex		
Male	1.50 (1.46-1.54)	1.37 (1.32-1.42)
Female	1.49 (1.45-1.53)	1.32 (1.27-1.38)

Abbreviations: aOR, adjusted odds ratio; ART, assisted reproductive technology; BMI, body mass index (calculated as weight in kilograms divided by height in meters squared).

^a^ Maternal age, race and ethnicity, educational level, marital status, parity, smoking status before pregnancy and during pregnancy, history of preterm delivery, history of cesarean delivery, prepregnancy BMI, timing of initiation of prenatal care, gestational hypertension, gestational diabetes, eclampsia, and neonate sex were adjusted for in the model except when the variable was a stratified variable. The natural conception group was the reference group.

^b^ Race and ethnicity data were self-reported in the database.

^c^ Other included non-Hispanic Native American or Alaska Native, non-Hispanic Asian, non-Hispanic Native Hawaiian or Other Pacific Islander, non-Hispanic people of more than 1 race, people of unknown racial or ethnic origin, or those who did not state race and ethnicity.

Sensitivity analyses, from which we excluded women with potential risk confounders such as cesarean delivery history, preterm delivery history, gestational hypertension or eclampsia, and gestational diabetes, yielded similar results as the main analyses (Table 4). After excluding these potential confounders simultaneously, the odds of preterm birth were 58% higher in the ART group (aOR, 1.58; 95% CI, 1.54-1.62) and 42% higher in the non-ART treatment group (aOR, 1.42; 95% CI, 1.37-1.47) compared with the natural conception group. The frequency of missing data was low for almost all covariates, but data for marital status was missing for 1 297 560 women (9.0%) (Table 1). In the sensitivity analysis that omitted participants with incomplete covariate data, the neonates who were conceived with the use of ART (aOR, 1.50; 95% CI, 1.47-1.53) or non-ART treatment (aOR,1.35; 95% CI, 1.31-1.39) had a higher risk of preterm birth (Table 4), which was consistent with the main results.

**Table 4.** Sensitivity Analysis for the Association of Fertility Treatment With Preterm Birth

Variable^a^	OR (95% CI)	
	ART group	Non-ART treatment group
Excluding women		
With cesarean delivery history	1.52 (1.49-1.55)	1.37 (1.33-1.41)
With preterm delivery history	1.52 (1.49-1.55)	1.37 (1.33-1.41)
With gestational hypertension or eclampsia	1.53 (1.49-1.56)	1.37 (1.33-1.41)
With gestational diabetes	1.51 (1.48-1.54)	1.37 (1.33-1.40)
Excluding women for cesarean delivery history, preterm delivery history, gestational hypertension or eclampsia, or gestational diabetes	1.58 (1.54-1.62)	1.42 (1.37-1.47)
With missing covariate data	1.50 (1.47-1.53)	1.35 (1.31-1.39)

Abbreviations: ART, assisted reproductive technology; OR, odds ratio.

^a^ Maternal age, race and ethnicity, educational level, marital status, parity, smoking status before pregnancy and during pregnancy, history of preterm delivery, history of cesarean delivery, prepregnancy body mass index, timing of initiation of prenatal care, gestational hypertension, gestational diabetes, eclampsia, and neonate sex were adjusted for in the model except when the variable was excluded. The natural conception group was the reference group.

## Discussion

In this nationwide population-based cohort study of more than 14 million multiracial and multiethnic pairs of mothers and singleton newborns, we found that conception using fertility treatment, either ART or non-ART, was significantly associated with preterm birth compared with natural conception after adjusting for all covariates. However, this result does not rule out the association of infertility with preterm delivery, and the risk outcome might be overestimated. Infertility is becoming a common health care problem and has drawn widespread attention in recent decades.^26^ Despite a large number of people worldwide who have conceived through fertility treatments, the potential adverse effects of these procedures, including preterm birth, have not been fully explored. We believe this study has added clinical and public health implications to the knowledge of fertility treatment outcomes.

The results of this study are consistent with those of existing studies that found an increased risk of preterm birth associated with fertility treatment–assisted pregnancies. Several cohort studies have compared the perinatal outcomes between singleton births using ART and singleton births from spontaneous conception, with adjustment for relevant confounders.^27,28^ A meta-analysis that included 8044 singleton births assisted by either in vitro fertilization or intracytoplasmic sperm injection and 53 633 singleton births by spontaneous conception found that the risk of preterm birth was significantly increased in ART-conceived singleton births.^18^ A cohort study in Finland reported that medically assisted reproduction, including ART and non-ART fertility drugs, was associated with an elevated risk of preterm birth outcomes.^29^ However, Martin et al^30^ found no association between increased risk of preterm birth and single-embryo transfer for singleton pregnancies, but double-embryo transfer was associated with a significant increase in the risk of preterm birth. This finding is not contrary to the results of the present study in that we did not conduct a subgroup analysis of detailed ART because of lack of proper data. A prospective cohort study that recruited 4252 women who conceived using ART observed no association between ART and preterm birth in singleton births.^19^ This result differed from the findings of this study, partly because of the limited sample size of the previous investigation.

We also analyzed the associations between non-ART treatment in singleton pregnancy and preterm birth. We found that non-ART treatment might be associated with an increased risk of preterm delivery after adjusting for all confounders. This result is partly consistent with findings from previous studies except that the subgroup analysis in the current study revealed a significant increase in the risk of extremely preterm, very preterm, and moderately preterm events. Wang et al^31^ reported that low reproduction technology, such as intrauterine insemination and donor insemination, was not associated with an increased risk of very preterm and extremely preterm birth, which contradicts the results of the present study. A potential reason for the difference might be the confounder of multiple pregnancies, and singleton pregnancies were the focus of this study.

Studies have found that women with previous preterm deliveries had a higher risk of preterm birth in their next pregnancy.^20,24^ However, in the stratified analyses, we observed no fertility treatment–associated increase in the risk of preterm birth among women with a history of preterm delivery. One explanation might be that women undergoing fertility treatment who have a history of preterm delivery might pay more attention to perinatal care, which could reduce the risk of preterm birth. Another possible explanation is that women with a history of preterm delivery are less likely to be infertile, and thus it might be reasonable to assume that the group with no history of preterm delivery comprises more women with infertility. Furthermore, in the sensitivity analyses, the results remained robust after excluding women with a history of preterm delivery.

Preterm birth in neonates who were conceived with fertility treatment is likely multifactorial. A major challenge for research that reports on this association is separating the contribution of infertility from that of ART. The fertility treatment may alter the endocrine profiles in pregnancy, which may affect placental and fetus development. Meanwhile, increasing evidence indicates that subfertility itself, including parental characteristics, may also have adverse implications for preterm birth.^32^ A meta-analysis of 7 studies reported an increased risk of preterm births associated with singleton births by spontaneous conception in women with subfertility compared with singleton births in fertile women.^33^ In the present study, we lacked the data to distinguish the association of infertility with preterm birth from the association of fertility treatment with preterm birth, and the risk associated with fertility treatment might be overestimated.

The potential mechanisms of the association between ART or non-ART treatment and preterm birth need to be elucidated. DNA methylation is a crucial element in the epigenetic regulation of mammalian embryonic development.^34^ Dysregulation of DNA methylation patterns associated with different ART methods may affect the development of placenta and fetus in the early embryonic stages and may introduce changes in growth patterns, and DNA methylation has already been found to contribute to the formation of congenital malformation.^35^ Although no manipulation of embryos was involved in non-ART treatment, the use of pharmacological agents to stimulate the production of oocytes was associated with poor-quality embryos.^36^ In addition, processing of sperm for artificial insemination might be associated with oxidative stress, which could potentially lead to chromosomal damage. Further research into the potential biological mechanisms of fertility treatment–induced preterm birth is warranted.

### Strengths and Limitations

This study has some strengths. It analyzed a nationwide population of mother-newborn pairs, included a large sample size, and had low levels of missing data, which provided sufficient statistical power to enable the examination of the association between maternal infertility treatment and preterm birth in subpopulations. Furthermore, the study excluded multiple pregnancies, pregestational hypertension, and pregestational diabetes. This approach limited confounding from these known preterm risk factors.

This study also has several limitations. First, a challenge in studying pregnancy outcomes in women who conceived by fertility treatment is selecting the appropriate comparison group. In this study, we used mothers with spontaneous conception as a reference. We assumed those who met the criteria of infertility while conceiving without fertility treatment would be a better comparison group. The 3 groups in this study (natural conception, ART, and non-ART) differed by age, socioeconomic status, educational level, and reproductive history, and we performed a series of logistic regression analyses and subgroup analyses to verify the reliability of the results. Second, with such an observational study, a central concern was unmeasured or uncontrolled confounding.^37,38^ In this study, mothers with fertility treatment tended to be older and were more likely to have gestational hypertension and gestational diabetes. Although we adjusted many maternal characteristics and complicated pregnancy conditions to mitigate confounding, we used administrative data and thus cannot guarantee complete and accurate data collection. We conducted a series of sensitivity analyses to verify the robustness of the main results, although residual confounding may still exist, and we could not ascertain that the adjustment was adequate. Furthermore, there were missing values for the variables included in the logistic regression analysis. However, a sensitivity analysis that deleted records with missing variables yielded similar results, suggesting the robustness of the data used in this study.

## Conclusions

This cohort study found that singleton births after fertility treatment had a higher risk of preterm birth. Understanding this risk is essential for individuals who are considering using fertility treatment to conceive and for physicians who provide prepregnancy and postpregnancy advice and care to these individuals. Further investigations are warranted into the mechanisms of the association between ART or non-ART treatment and the risk of preterm birth.

## Notes

Supplement. **eTable 1.** The Baseline Comparison Between Included and Excluded Participants **eTable 2.** Multivariable Logistic Regression of Odds of Fertility Treatment and Preterm Birth: Full Regression Results
