# Supplementary material for: Association of Preterm Singleton Birth With Fertility Treatment in the US
Source: JAMA Netw Open. 2022 Feb 8;5(2):e2147782. doi: 10.1001/jamanetworkopen.2021.47782 (PMC8826170; doi:10.1001/jamanetworkopen.2021.47782)
Supplement: Supplement. — eTable 1. The Baseline Comparison Between Included and Excluded Participants eTable 2. Multivariable Logistic Regression of Odds of Fertility Treatment and Preterm Birth: Full Regression Results [file jamanetwopen-e2147782-s001.pdf]

Supplementary Online Content

Wang R, Shi Q, Jia B, et al. Association of preterm singleton birth with fertility treatment in the US. *JAMA Netw Open*. 2022;5(2):e2147782.
doi:10.1001/jamanetworkopen.2021.47782

eTable 1. The Baseline Comparison Between Included and Excluded Participants

eTable 2. Multivariable Logistic Regression of Odds of Fertility Treatment and Preterm Birth: Full Regression Results

This supplementary material has been provided by the authors to give readers additional information about their work.

© 2022 Wang R et al. *JAMA Network Open.*

eTable 1. The Baseline Comparison Between Included and Excluded Participants

	All	Excluded	Included	P
Number, n (%)	15379982 (100.00)	1009062 (6.56)	14370920 (93.44)	/
Birth year, n (%)				<0.001
2016	3704404 (25.78)	251708 (24.94)	3,704,404 (25.78)	
2017	3612116 (25.13)	252638 (25.04)	3,612,116 (25.13)	
2018	3549151 (24.70)	252383 (25.01)	3,549,151 (24.70)	
2019	3505249 (24.39)	252333 (25.01)	3,505,249 (24.39)	
Age (y), mean (SD）	28.92 (5.82)	30.67 (5.88)	28.79 (5.79)	<0.001
Age group (y), n (%)				<0.001
<30	8231948 (53.52)	422179 (41.84)	7809769 (54.34)	
30-35	4395132 (28.58)	315080 (31.23)	4080052 (28.39)	
35-39	2247936 (14.62)	208821 (20.69)	2039115 (14.19)	
≥40	504966 (3.28)	62982 (6.24)	441984 (3.08)	
Race/ethnicity, n (%)				<0.001
Hispanic	3620257 (23.54)	186393 (18.47)	3433864 (23.89)	
non-Hispanic white	7924918 (51.53)	512786 (50.82)	7412132 (51.58)	
non-Hispanic black	2221881 (14.45)	209318 (20.74)	2012563 (14.00)	
Other*	1473639 (9.58)	86100 (8.53)	1387539 (9.66)	
missing	139287 (0.91)	14465 (1.43)	124822 (0.87)	
Education level, n (%)				<0.001
lower than high school	1978254 (12.86)	112894 (11.19)	1865360 (12.98)	
high school	3891505 (25.30)	243168 (24.10)	3648337 (25.39)	
higher than high school	9304987 (60.50)	632090 (62.64)	8672897 (60.35)	
missing	205236 (1.33)	20910 (2.07)	184326 (1.28)	
Marital status, n(%)				<0.001
married	8398429 (54.61)	564963 (55.99)	7833466 (54.51)	
unmarried	5604997 (36.44)	365103 (36.18)	5239894 (36.46)	
missing	1376556 (8.95)	78996 (7.83)	1297560 (9.03)	
Parity, n (%)				<0.001
0	5838507 (37.96)	298786 (29.61)	5539721 (38.55)	
1	4905329 (31.89)	314709 (31.19)	4590620 (31.94)	
2	2629398 (17.10)	199665 (19.79)	2429733 (16.91)	
3	1142425 (7.43)	101932 (10.10)	1040493 (7.24)	
≥4	819082 (5.33)	85934 (8.52)	733148 (5.10)	
missing	45241 (0.29)	8036 (0.80)	37205 (0.26)	
Smoking before pregnancy, n (%)				<0.001
Yes	1319997 (8.58)	96599 (9.57)	1223398 (8.51)	
No	13983845 (90.92)	903240 (89.51)	13080605 (91.02)	
missing	76140 (0.50)	9223 (0.91)	66917 (0.47)	
Smoking during pregnancy, n (%)				<0.001
Yes	1015269 (6.60)	75059 (7.44)	940210 (6.54)	

© 2022 Wang R et al. *JAMA Network Open.*

No	14254360 (92.68)	907234 (89.91)	13347126 (92.88)	
missing	110353 (0.72)	26769 (2.65)	83584 (0.58)	
Time of iniation of prenatal care, n (%)				<0.001
no prenatal care	262151 (1.70)	25024 (2.48)	237127 (1.65)	
1st-3rd month	11585600 (75.33)	763197 (75.63)	10822403 (75.31)	
4th-6th month	2455920 (15.97)	148245 (14.69)	2307675 (16.06)	
7th-final month	683316 (4.44)	32240 (3.20)	651076 (4.53)	
missing	392995 (2.56)	40356 (4.00)	352639 (2.45)	
Prepregnancy BMI (kg/m2), mean (SD)	28.86 (13.11)	32.56 (15.40)	28.60 (12.89)	
Prepregnancy BMI (kg/m2), n (%)				<0.001
<18.5	492166 (3.20)	18272 (1.81)	473894 (3.30)	
18.5-24.9	6411728 (41.69)	295324 (29.27)	6116404 (42.56)	
25.0-29.9	3967727 (25.80)	239199 (23.71)	3728528 (25.94)	
30.0-34.9	2238474 (14.55)	180249 (17.86)	2058225 (14.32)	
35.0-39.9	1102005 (7.17)	117110 (11.61)	984895 (6.85)	
≥40	788339 (5.13)	122114 (12.10)	666225 (4.64)	
missing	379543 (2.47)	36794 (3.65)	342749 (2.39)	
Gestational hypertention, n (%)				<0.001
Yes	1048800 (6.82)	95531 (9.47)	953269 (6.63)	
No	14317119 (93.09)	899468 (89.14)	13417651 (93.37)	
missing	14063 (0.09)	14063 (1.39)	/	
Gestational diabetes, n (%)				<0.001
Yes	992141 (6.45)	92736 (9.19)	899405 (6.26)	
No	14373778 (93.46)	902263 (89.42)	13471515 (93.74)	
missing	14063 (0.09)	14063 (1.39)	/	
Eclampsia, n (%)				<0.001
Yes	41083 (0.27)	7227 (0.72)	33856 (0.24)	
No	15324836 (99.64)	987772 (97.89)	14337064 (99.76)	
missing	14063 (0.09)	14063 (1.39)	/	
Previous history of preterm birth, n (%)				<0.001
Yes	519597 (3.38)	66451 (6.59)	453146 (3.15)	
No	14846322 (96.53)	928548 (92.02)	13917774 (96.85)	
missing	14063 (0.09)	14063 (1.39)		
Previous history of Cesarean, n (%)				<0.001
Yes	2383398 (15.50)	201123 (19.93)	2182275 (15.19)	
No	12982521 (84.41)	793876 (78.67)	12188645 (84.81)	
missing	14063 (0.09)	14063 (1.39)	/	
Birth weight (g), n (%)				<0.001
<1000	99934 (0.65)	48111 (4.77)	51823 (0.36)	
1000-1500	113547 (0.74)	41669 (4.13)	71878 (0.50)	
1500-2499	1054649 (6.86)	299462 (29.68)	755187 (5.25)	
>=2500	14097549 (91.66)	611565 (60.61)	13485984 (93.84)	

© 2022 Wang R et al. *JAMA Network Open.*

missing	14303 (0.09)	8255 (0.82)	6048 (0.04)	
Infant sex, n (%)				0.003
Male	7867059 (51.15)	514695 (51.01)	7352364 (51.16)	
Female	7512923 (48.85)	494367 (48.99)	7018556 (48.84)	
Preterm birth, n(%)				<0.001
No	13832046 (89.94)	552954 (54.80)	13279092 (92.40)	
Yes	1536487 (9.99)	444659 (44.07)	1091828 (7.60)	
extremely preterm birth	102390 (0.67)	50149 (4.97)	52241 (0.36)	
very preterm birth	141063 (0.92)	50190 (4.97)	90873 (0.63)	
moderate and late preterm birth	1293034 (8.41)	344320 (34.12)	948714 (6.60)	
missing	11449 (0.07)	11449 (1.13)	/	
Fertility treatment, n(%)				<0.001
Natural conception	15074516 (98.01)	897716 (88.97)	14176800 (98.65)	
ART treatment	176219 (1.15)	53275 (5.28)	122944 (0.86)	
Non-ART treatment	96667 (0.63)	25491 (2.53)	71176 (0.50)	
missing	32580 (0.21)	32580 (3.23)	/	

*Included non-Hispanic Native American or Alaskans, non-Hispanic Asians, non-Hispanic Native Hawaiians or other Pacific Islanders, non-Hispanic people of more than one race, people of unknown racial or ethnic origin, or not stated.

© 2022 Wang R et al. *JAMA Network Open.*

eTable 2. Multivariable Logistic Regression of Odds of Fertility Treatment and Preterm Birth: Full Regression Results

	aOR (95%CI)
Fertility treatment	
natural conception group	reference
ART group	1.49 (1.46, 1.52)
non-ART group	1.35 (1.31, 1.38)
mother age (y)	1.0126(1.0122, 1.0133)
Race/ethnicity	
Hispanic	reference
non-Hispanic white	0.871 (0.867, 0.876)
non-Hispanic black	1.25 (1.24, 1.26)
Other*	0.985 (0.977, 0.993)
missing	0.93 (0.90, 0.95)
Education level	
lower than high school	reference
high school	0.952 (0.946, 0.958)
higher than high school	0.835 (0.829, 0.840)
missing	0.98 (0.96, 1.00)
Marital status	
married	reference
unmarried	1.22 (1.21, 1.23)
missing	1.03 (1.02, 1.04)
Parity, (n)	
0	reference
1	0.766 (0.762, 0.770)
2	0.800 (0.795, 0.806)
3	0.877 (0.870, 0.884)
≥4	0.938 (0.929, 0.946)
missing	0.828 (0.799, 0.858)
Smoking before pregnancy	
No	reference
Yes	0.92 (0.91, 0.93)
missing	0.022 (0.021, 0.023)
Smoking during pregnancy	
No	reference
Yes	1.50 (1.57, 1.61)
missing	62.06 (59.71, 64.50)
Time of iniation of prenatal care	
no prenatal care	reference
1st-3rd month	0.330 (0.326, 0.333)
4th-6th month	0.291 (0.2880, 0.295)

© 2022 Wang R et al. *JAMA Network Open.*

7th-final month	0.248 (0.245, 0.252)
missing	0.598 (0.590, 0.607)
Prepregnancy BMI (kg/m2),	1.0019 (1.0018, 1.0021)
Gestational hypertention	
No	reference
Yes	2.96 (2.94, 2.98)
Gestational diabetes	
No	reference
Yes	1.34 (1.33, 1.35)
Eclampsia	
No	reference
Yes	5.41 (5.28, 5.54)
Previous history of preterm birth	
No	reference
Yes	4.09 (4.06, 4.12)
Previous history of Cesarean	
No	reference
Yes	1.21 (1.20, 1.22)

*Included non-Hispanic Native American or Alaskans, non-Hispanic Asians, non-Hispanic Native Hawaiians or other Pacific Islanders, non-Hispanic people of more than one race, people of unknown racial or ethnic origin, or not stated.

© 2022 Wang R et al. *JAMA Network Open.*
